# Supplementary material for: Characterization of shifts of koala (Phascolarctos cinereus) intestinal microbial communities associated with antibiotic treatment
Source: PeerJ. 2018 Mar 12;6:e4452. doi: 10.7717/peerj.4452 (PMC5853612; doi:10.7717/peerj.4452)
Supplement: Table S1 [file peerj-06-4452-s001.docx]

| **OTU** | **Domain** | **Phylum** | **Class** | **Order** | **Family** | **Genus** | **Species** |
| --- | --- | --- | --- | --- | --- | --- | --- |
| *2496* | *Bacteria* | *Proteobacteria* | *Gammaproteobacteria* | *Pasteurellales* | *Pasteurellaceae* | *Lonepinella* | *koalarum* |
| *2259* | *Bacteria* | *Proteobacteria* | *Gammaproteobacteria* | *Pasteurellales* | *Pasteurellaceae* | *Lonepinella* | *koalarum* |
| *1706* | *Bacteria* | *Firmicutes* | *Clostridia* | *Clostridiales* | *Ruminococcaceae* |  |  |
| *370* | *Bacteria* | *Proteobacteria* | *Gammaproteobacteria* | *Pasteurellales* | *Pasteurellaceae* | *Lonepinella* | *koalarum* |
| *2358* | *Bacteria* | *Cyanobacteria* | *4C0d-2* | *YS2* |  |  |  |
| *2297* | *Bacteria* | *Bacteroidetes* | *Bacteroidia* | *Bacteroidales* | *Porphyromonadaceae* |  |  |
| *1705* | *Bacteria* | *Bacteroidetes* | *Bacteroidia* | *Bacteroidales* | *Bacteroidaceae* | *Bacteroides* | *fragilis* |
| *122* | *Bacteria* | *Proteobacteria* | *Gammaproteobacteria* | *Pasteurellales* | *Pasteurellaceae* | *Lonepinella* | *koalarum* |
| *1027* | *Bacteria* | *Firmicutes* | *Clostridia* | *Clostridiales* | *Ruminococcaceae* | *Ruminococcus* | *flavefaciens* |
| *2185* | *Bacteria* | *Bacteroidetes* | *Bacteroidia* | *Bacteroidales* | *Bacteroidaceae* | *Bacteroides* | *fragilis* |
| *1463* | *Bacteria* | *Firmicutes* | *Clostridia* | *Clostridiales* | *Ruminococcaceae* |  |  |
| *2275* | *Bacteria* | *Proteobacteria* | *Gammaproteobacteria* | *Pasteurellales* | *Pasteurellaceae* |  |  |
| *1562* | *Bacteria* | *Proteobacteria* | *Gammaproteobacteria* | *Pasteurellales* | *Pasteurellaceae* | *Lonepinella* | *koalarum* |
| *1855* | *Bacteria* | *Verrucomicrobia* | *Verrucomicrobiae* | *Verrucomicrobiales* | *Verrucomicrobiaceae* | *Akkermansia* | *muciniphila* |
| *2366* | *Bacteria* | *Bacteroidetes* | *Bacteroidia* | *Bacteroidales* | *Rikenellaceae* |  |  |
| *4499* | *Bacteria* | *Firmicutes* | *Clostridia* | *Clostridiales* | *Ruminococcaceae* |  |  |
| *170* | *Bacteria* | *Bacteroidetes* | *Bacteroidia* | *Bacteroidales* | *Bacteroidaceae* | *Bacteroides* | *fragilis* |
| *2503* | *Bacteria* | *Bacteroidetes* | *Bacteroidia* | *Bacteroidales* | *Bacteroidaceae* | *Bacteroides* |  |
| *2390* | *Bacteria* | *Firmicutes* | *Clostridia* | *Clostridiales* | *Ruminococcaceae* | *Ruminococcus* | *flavefaciens* |
| *1450* | *Bacteria* | *Proteobacteria* | *Deltaproteobacteria* | *Desulfobacterales* | *Desulfobacteraceae* |  |  |
| *445* | *Bacteria* | *Firmicutes* | *Clostridia* | *Clostridiales* | *Ruminococcaceae* |  |  |
| *2363* | *Bacteria* | *Proteobacteria* | *Gammaproteobacteria* | *Pasteurellales* | *Pasteurellaceae* | *Lonepinella* | *koalarum* |
| *272* | *Bacteria* | *Proteobacteria* | *Gammaproteobacteria* | *Pasteurellales* | *Pasteurellaceae* | *Lonepinella* | *koalarum* |
| *1474* | *Bacteria* | *Bacteroidetes* | *Bacteroidia* | *Bacteroidales* | *Bacteroidaceae* | *Bacteroides* | *uniformis* |
| *169* | *Bacteria* | *Proteobacteria* | *Alphaproteobacteria* | *Rhizobiales* | *Methylobacteriaceae* | *Methylobacterium* |  |
| *1011* | *Bacteria* | *Proteobacteria* | *Gammaproteobacteria* | *Pasteurellales* | *Pasteurellaceae* | *Lonepinella* | *koalarum* |
| *2236* | *Bacteria* | *Bacteroidetes* | *Bacteroidia* | *Bacteroidales* | *Bacteroidaceae* | *Bacteroides* | *fragilis* |
| *2310* | *Bacteria* | *Firmicutes* | *Clostridia* | *Clostridiales* | *Veillonellaceae* |  |  |
| *1693* | *Bacteria* | *Proteobacteria* | *Deltaproteobacteria* | *Desulfovibrionales* | *Desulfovibrionaceae* |  |  |
| *1231* | *Bacteria* | *Proteobacteria* | *Deltaproteobacteria* | *Desulfobacterales* | *Desulfobacteraceae* |  |  |
| *2346* | *Bacteria* | *Proteobacteria* | *Gammaproteobacteria* | *Pasteurellales* | *Pasteurellaceae* | *Lonepinella* | *koalarum* |
| *2256* | *Bacteria* | *Proteobacteria* | *Deltaproteobacteria* | *Desulfovibrionales* | *Desulfovibrionaceae* |  |  |
| *2189* | *Bacteria* | *Planctomycetes* | *vadinHA49* | *PeHg47* |  |  |  |
| *537* | *Bacteria* | *Proteobacteria* | *Gammaproteobacteria* | *Pasteurellales* | *Pasteurellaceae* | *Lonepinella* | *koalarum* |
| *500* | *Bacteria* | *Bacteroidetes* | *Bacteroidia* | *Bacteroidales* | *Bacteroidaceae* | *Bacteroides* | *fragilis* |
| *355* | *Bacteria* | *Firmicutes* | *Clostridia* | *Clostridiales* | *Ruminococcaceae* | *Ruminococcus* |  |
| *2331* | *Bacteria* | *Proteobacteria* | *Gammaproteobacteria* | *Pasteurellales* | *Pasteurellaceae* | *Lonepinella* | *koalarum* |
| *1330* | *Bacteria* | *Proteobacteria* | *Deltaproteobacteria* | *Desulfovibrionales* | *Desulfovibrionaceae* |  |  |
| *158* | *Bacteria* | *Bacteroidetes* | *Bacteroidia* | *Bacteroidales* | *Bacteroidaceae* | *Bacteroides* | *fragilis* |
| *2442* | *Bacteria* | *Firmicutes* | *Clostridia* | *Clostridiales* | *Ruminococcaceae* |  |  |
| *2183* | *Bacteria* | *Firmicutes* | *Clostridia* | *Clostridiales* | *Ruminococcaceae* |  |  |
| *338* | *Bacteria* | *Bacteroidetes* | *Bacteroidia* | *Bacteroidales* | *Bacteroidaceae* | *Bacteroides* | *fragilis* |
| *2430* | *Bacteria* | *Firmicutes* | *Clostridia* | *Clostridiales* | *Ruminococcaceae* | *Oscillospira* |  |
| *922* | *Bacteria* | *Firmicutes* | *Clostridia* | *Clostridiales* | *Ruminococcaceae* | *Ruminococcus* | *flavefaciens* |
| *112* | *Bacteria* | *Firmicutes* | *Clostridia* | *Clostridiales* | *Ruminococcaceae* | *Ruminococcus* |  |
| *1882* | *Bacteria* | *Proteobacteria* | *Deltaproteobacteria* | *Desulfovibrionales* | *Desulfovibrionaceae* | *Desulfovibrio* |  |
| *87* | *Bacteria* | *Firmicutes* | *Clostridia* | *Clostridiales* | *Ruminococcaceae* |  |  |
| *2261* | *Bacteria* | *Firmicutes* | *Clostridia* | *Clostridiales* | *Ruminococcaceae* |  |  |
| *2161* | *Bacteria* | *Bacteroidetes* | *Bacteroidia* | *Bacteroidales* | *Bacteroidaceae* | *Bacteroides* |  |
| *4619* | *Bacteria* | *Bacteroidetes* | *Bacteroidia* | *Bacteroidales* | *Porphyromonadaceae* | *Parabacteroides* |  |
| *1126* | *Bacteria* | *Proteobacteria* | *Deltaproteobacteria* | *Desulfovibrionales* | *Desulfovibrionaceae* |  |  |
| *467* | *Bacteria* | *Firmicutes* | *Clostridia* | *Clostridiales* | *Ruminococcaceae* | *Ruminococcus* | *flavefaciens* |
| *2319* | *Bacteria* | *Bacteroidetes* | *Bacteroidia* | *Bacteroidales* | *Porphyromonadaceae* | *Paludibacter* |  |
| *2399* | *Bacteria* | *Cyanobacteria* | *4C0d-2* | *YS2* |  |  |  |
| *2274* | *Bacteria* | *Bacteroidetes* | *Bacteroidia* | *Bacteroidales* | *Porphyromonadaceae* | *Parabacteroides* |  |
| *2364* | *Bacteria* | *Proteobacteria* | *Betaproteobacteria* | *Rhodocyclales* | *Rhodocyclaceae* |  |  |
| *2428* | *Bacteria* | *Firmicutes* | *Clostridia* | *Clostridiales* |  |  |  |
| *1878* | *Bacteria* | *Actinobacteria* | *Actinobacteria* | *Actinomycetales* | *Micrococcaceae* | *Kocuria* | *palustris* |
| *2248* | *Bacteria* | *Firmicutes* | *Clostridia* | *Clostridiales* |  |  |  |
| *346* | *Bacteria* | *Proteobacteria* | *Gammaproteobacteria* | *Pseudomonadales* | *Pseudomonadaceae* | *Pseudomonas* |  |
| *2473* | *Bacteria* | *Firmicutes* | *Clostridia* | *Clostridiales* | *Veillonellaceae* |  |  |
| *2373* | *Bacteria* | *Cyanobacteria* | *4C0d-2* | *YS2* |  |  |  |
| *2354* | *Bacteria* | *Firmicutes* | *Clostridia* | *Clostridiales* | *Ruminococcaceae* | *Oscillospira* |  |
| *2280* | *Bacteria* | *Bacteroidetes* | *Bacteroidia* | *Bacteroidales* | *Porphyromonadaceae* | *Candidatus Azobacteroides* |  |
| *1465* | *Bacteria* | *Firmicutes* | *Clostridia* | *Clostridiales* | *Lachnospiraceae* |  |  |
| *867* | *Bacteria* | *Bacteroidetes* | *Bacteroidia* | *Bacteroidales* | *Bacteroidaceae* | *Bacteroides* | *uniformis* |
| *4636* | *Bacteria* | *Proteobacteria* | *Deltaproteobacteria* | *Desulfovibrionales* | *Desulfovibrionaceae* |  |  |
| *1044* | *Bacteria* | *Firmicutes* | *Clostridia* | *Clostridiales* | *Lachnospiraceae* | *Dorea* |  |
| *1868* | *Bacteria* | *Proteobacteria* | *Deltaproteobacteria* | *Desulfovibrionales* | *Desulfovibrionaceae* |  |  |
| *2322* | *Bacteria* | *Bacteroidetes* | *Bacteroidia* | *Bacteroidales* | *Bacteroidaceae* | *Bacteroides* |  |
| *852* | *Bacteria* | *Firmicutes* | *Clostridia* | *Clostridiales* | *Ruminococcaceae* |  |  |
| *1333* | *Bacteria* | *Bacteroidetes* | *Bacteroidia* | *Bacteroidales* | *Bacteroidaceae* | *Bacteroides* | *fragilis* |
| *4858* | *Bacteria* | *Firmicutes* | *Clostridia* | *Clostridiales* | *Lachnospiraceae* | *Dorea* |  |
| *222* | *Bacteria* | *Firmicutes* | *Clostridia* | *Clostridiales* | *Ruminococcaceae* | *Oscillospira* |  |
| *2307* | *Bacteria* | *Bacteroidetes* | *Bacteroidia* | *Bacteroidales* | *Porphyromonadaceae* | *Tannerella* |  |
| *2042* | *Bacteria* | *Firmicutes* | *Clostridia* | *Clostridiales* | *Ruminococcaceae* |  |  |
| *5924* | *Bacteria* | *Firmicutes* | *Clostridia* | *Clostridiales* | *Ruminococcaceae* |  |  |
| *2149* | *Bacteria* | *Firmicutes* | *Clostridia* | *Clostridiales* |  |  |  |
| *3089* | *Bacteria* | *Firmicutes* | *Clostridia* | *Clostridiales* | *Ruminococcaceae* |  |  |
| *2455* | *Bacteria* | *Firmicutes* | *Clostridia* | *Clostridiales* | *Ruminococcaceae* |  |  |
| *1325* | *Bacteria* | *Firmicutes* | *Clostridia* | *Clostridiales* | *Ruminococcaceae* | *Ruminococcus* | *flavefaciens* |
| *594* | *Bacteria* | *Fusobacteria* | *Fusobacteriia* | *Fusobacteriales* | *Fusobacteriaceae* | *Fusobacterium* |  |
| *1400* | *Bacteria* | *Proteobacteria* | *Deltaproteobacteria* | *Desulfarculales* | *Desulfarculaceae* |  |  |
| *2435* | *Bacteria* | *Bacteroidetes* | *Bacteroidia* | *Bacteroidales* | *Bacteroidaceae* | *Bacteroides* | *uniformis* |
| *2167* | *Bacteria* | *Bacteroidetes* | *Bacteroidia* | *Bacteroidales* | *Bacteroidaceae* | *Bacteroides* | *fragilis* |
| *1282* | *Bacteria* | *Bacteroidetes* | *Bacteroidia* | *Bacteroidales* | *Bacteroidaceae* | *Bacteroides* |  |
| *1531* | *Bacteria* | *Cyanobacteria* | *4C0d-2* | *YS2* |  |  |  |
| *2172* | *Bacteria* | *Bacteroidetes* | *Bacteroidia* | *Bacteroidales* | *Porphyromonadaceae* | *Parabacteroides* | *distasonis* |
| *2494* | *Bacteria* | *Bacteroidetes* | *Bacteroidia* | *Bacteroidales* | *Bacteroidaceae* | *Bacteroides* |  |
| *1750* | *Bacteria* | *Firmicutes* | *Clostridia* | *Clostridiales* |  |  |  |
| *1867* | *Bacteria* | *Bacteroidetes* | *Bacteroidia* | *Bacteroidales* | *Bacteroidaceae* | *Bacteroides* | *fragilis* |
| *681* | *Bacteria* | *Proteobacteria* | *Deltaproteobacteria* | *Desulfovibrionales* | *Desulfovibrionaceae* |  |  |
| *2464* | *Bacteria* | *Bacteroidetes* | *Bacteroidia* | *Bacteroidales* | *Porphyromonadaceae* | *Parabacteroides* |  |
| *2432* | *Bacteria* | *Synergistetes* | *Synergistia* | *Synergistales* | *Synergistaceae* |  |  |
| *4864* | *Bacteria* | *Firmicutes* | *Clostridia* | *Clostridiales* | *Ruminococcaceae* |  |  |
| *1423* | *Bacteria* | *Firmicutes* | *Clostridia* | *Clostridiales* | *Ruminococcaceae* | *Oscillospira* |  |
| *2470* | *Bacteria* | *Cyanobacteria* | *4C0d-2* | *YS2* |  |  |  |
| *2121* | *Bacteria* | *Bacteroidetes* | *Bacteroidia* | *Bacteroidales* | *Bacteroidaceae* | *Bacteroides* | *fragilis* |
| *2414* | *Bacteria* | *Firmicutes* | *Clostridia* | *Clostridiales* |  |  |  |
| *2272* | *Bacteria* | *Bacteroidetes* | *Bacteroidia* | *Bacteroidales* | *Porphyromonadaceae* | *Parabacteroides* |  |
| *332* | *Bacteria* | *Proteobacteria* | *Deltaproteobacteria* | *Desulfarculales* | *Desulfarculaceae* |  |  |
| *1010* | *Bacteria* | *Proteobacteria* | *Gammaproteobacteria* | *Pasteurellales* | *Pasteurellaceae* | *Lonepinella* | *koalarum* |
| *2252* | *Bacteria* | *Bacteroidetes* | *Bacteroidia* | *Bacteroidales* | *Rikenellaceae* |  |  |
| *3579* | *Bacteria* | *Firmicutes* | *Clostridia* | *Clostridiales* | *Ruminococcaceae* | *Ruminococcus* |  |
| *1137* | *Bacteria* | *Bacteroidetes* | *Bacteroidia* | *Bacteroidales* | *Bacteroidaceae* | *Bacteroides* | *fragilis* |
| *2360* | *Bacteria* | *Bacteroidetes* | *Bacteroidia* | *Bacteroidales* | *Porphyromonadaceae* | *Parabacteroides* |  |
| *3529* | *Bacteria* | *Proteobacteria* | *Deltaproteobacteria* | *Desulfovibrionales* | *Desulfovibrionaceae* | *Bilophila* |  |
| *2245* | *Bacteria* | *Firmicutes* | *Clostridia* | *Clostridiales* | *Ruminococcaceae* |  |  |
| *3794* | *Bacteria* | *Firmicutes* | *Clostridia* | *Clostridiales* | *Ruminococcaceae* |  |  |
| *2112* | *Bacteria* | *Firmicutes* | *Clostridia* | *Clostridiales* | *Lachnospiraceae* | *Blautia* | *producta* |
| *4298* | *Bacteria* | *Firmicutes* | *Clostridia* | *Clostridiales* |  |  |  |
| *2403* | *Bacteria* | *Fusobacteria* | *Fusobacteriia* | *Fusobacteriales* | *Fusobacteriaceae* | *Fusobacterium* |  |
| *16* | *Bacteria* | *Proteobacteria* | *Alphaproteobacteria* | *Sphingomonadales* | *Sphingomonadaceae* | *Sphingomonas* |  |
| *93* | *Bacteria* | *Bacteroidetes* | *Bacteroidia* | *Bacteroidales* | *Bacteroidaceae* | *Bacteroides* | *fragilis* |
| *395* | *Bacteria* | *Firmicutes* | *Clostridia* | *Clostridiales* | *Ruminococcaceae* | *Ruminococcus* | *flavefaciens* |
| *2273* | *Bacteria* | *Firmicutes* | *Clostridia* | *Clostridiales* | *Ruminococcaceae* |  |  |
| *2404* | *Bacteria* | *Synergistetes* | *Synergistia* | *Synergistales* | *Synergistaceae* |  |  |
| *2475* | *Bacteria* | *Proteobacteria* | *Gammaproteobacteria* | *Pasteurellales* | *Pasteurellaceae* | *Lonepinella* | *koalarum* |
| *1655* | *Bacteria* | *Bacteroidetes* | *Bacteroidia* | *Bacteroidales* | *Bacteroidaceae* | *Bacteroides* |  |
| *92* | *Bacteria* | *Fusobacteria* | *Fusobacteriia* | *Fusobacteriales* | *Fusobacteriaceae* | *Fusobacterium* |  |
| *1250* | *Bacteria* | *Bacteroidetes* | *Bacteroidia* | *Bacteroidales* | *Bacteroidaceae* | *Bacteroides* |  |
| *2385* | *Bacteria* | *Proteobacteria* | *Gammaproteobacteria* | *Pasteurellales* | *Pasteurellaceae* | *Lonepinella* | *koalarum* |
| *2437* | *Bacteria* | *Firmicutes* | *Clostridia* | *Clostridiales* | *Ruminococcaceae* | *Ruminococcus* |  |
| *5421* | *Bacteria* | *Firmicutes* | *Clostridia* | *Clostridiales* | *Ruminococcaceae* |  |  |
| *2909* | *Bacteria* | *Proteobacteria* | *Deltaproteobacteria* | *Desulfovibrionales* | *Desulfovibrionaceae* |  |  |
| *3483* | *Bacteria* | *Firmicutes* | *Erysipelotrichi* | *Erysipelotrichales* | *Erysipelotrichaceae* | *Coprobacillus* |  |
| *2458* | *Bacteria* | *Firmicutes* | *Clostridia* | *Clostridiales* | *Ruminococcaceae* |  |  |
| *783* | *Bacteria* | *Bacteroidetes* | *Bacteroidia* | *Bacteroidales* | *Bacteroidaceae* | *Bacteroides* | *fragilis* |
| *2965* | *Bacteria* | *Synergistetes* | *Synergistia* | *Synergistales* | *Synergistaceae* |  |  |
| *2134* | *Bacteria* | *Bacteroidetes* | *Bacteroidia* | *Bacteroidales* | *Bacteroidaceae* | *Bacteroides* | *fragilis* |
| *2383* | *Bacteria* | *Bacteroidetes* | *Bacteroidia* | *Bacteroidales* | *Bacteroidaceae* | *Bacteroides* |  |
| *2296* | *Bacteria* | *Firmicutes* | *Clostridia* | *Clostridiales* | *Veillonellaceae* |  |  |
| *546* | *Bacteria* | *Bacteroidetes* | *Bacteroidia* | *Bacteroidales* | *Porphyromonadaceae* | *Parabacteroides* | *distasonis* |
| *1560* | *Bacteria* | *Bacteroidetes* | *Bacteroidia* | *Bacteroidales* | *Bacteroidaceae* | *Bacteroides* |  |
| *1747* | *Bacteria* | *Bacteroidetes* | *Bacteroidia* | *Bacteroidales* | *Rikenellaceae* |  |  |
| *453* | *Bacteria* | *Firmicutes* | *Clostridia* | *Clostridiales* |  |  |  |
| *2157* | *Bacteria* | *Bacteroidetes* | *Bacteroidia* | *Bacteroidales* | *Porphyromonadaceae* | *Parabacteroides* |  |
| *469* | *Bacteria* | *Firmicutes* | *Clostridia* | *Clostridiales* | *Lachnospiraceae* |  |  |
| *3551* | *Bacteria* | *Proteobacteria* | *Betaproteobacteria* | *Rhodocyclales* | *Rhodocyclaceae* | *Petrobacter* | *succinatimandens* |
| *1362* | *Bacteria* | *Proteobacteria* | *Deltaproteobacteria* | *Desulfovibrionales* | *Desulfovibrionaceae* |  |  |
| *2421* | *Bacteria* | *Bacteroidetes* | *Bacteroidia* | *Bacteroidales* | *Porphyromonadaceae* | *Parabacteroides* |  |
| *1812* | *Bacteria* | *Bacteroidetes* | *Bacteroidia* | *Bacteroidales* | *Bacteroidaceae* | *Bacteroides* |  |
| *2461* | *Bacteria* | *Firmicutes* | *Clostridia* | *Clostridiales* | *Ruminococcaceae* | *Ruminococcus* |  |
| *2278* | *Bacteria* | *Proteobacteria* | *Gammaproteobacteria* | *Pasteurellales* | *Pasteurellaceae* | *Lonepinella* | *koalarum* |
| *1923* | *Bacteria* | *Proteobacteria* | *Deltaproteobacteria* | *Desulfovibrionales* | *Desulfovibrionaceae* | *Bilophila* |  |
| *2481* | *Bacteria* | *Proteobacteria* | *Deltaproteobacteria* | *Desulfovibrionales* | *Desulfovibrionaceae* |  |  |
| *2309* | *Bacteria* | *Bacteroidetes* | *Bacteroidia* | *Bacteroidales* | *Porphyromonadaceae* | *Parabacteroides* |  |
| *2339* | *Bacteria* | *Cyanobacteria* | *4C0d-2* | *YS2* |  |  |  |
| *1340* | *Bacteria* | *Firmicutes* | *Bacilli* | *Lactobacillales* | *Streptococcaceae* | *Streptococcus* |  |
| *1610* | *Bacteria* | *Verrucomicrobia* | *Verrucomicrobiae* | *Verrucomicrobiales* | *Verrucomicrobiaceae* | *Akkermansia* | *muciniphila* |
| *1192* | *Bacteria* | *Proteobacteria* | *Gammaproteobacteria* | *Pasteurellales* | *Pasteurellaceae* | *Lonepinella* | *koalarum* |
| *767* | *Bacteria* | *Bacteroidetes* | *Bacteroidia* | *Bacteroidales* | *Porphyromonadaceae* | *Parabacteroides* | *distasonis* |
| *544* | *Bacteria* | *Firmicutes* | *Clostridia* | *Clostridiales* | *Lachnospiraceae* |  |  |
| *2282* | *Bacteria* | *Bacteroidetes* | *Bacteroidia* | *Bacteroidales* | *Rikenellaceae* |  |  |
| *133* | *Bacteria* | *Firmicutes* | *Clostridia* | *Clostridiales* | *Lachnospiraceae* |  |  |
| *2317* | *Bacteria* | *Proteobacteria* | *Betaproteobacteria* | *Burkholderiales* | *Alcaligenaceae* | *Sutterella* |  |
| *2492* | *Bacteria* | *Firmicutes* | *Clostridia* | *Clostridiales* |  |  |  |
| *3174* | *Bacteria* | *Fusobacteria* | *Fusobacteriia* | *Fusobacteriales* | *Fusobacteriaceae* | *Fusobacterium* |  |
| *1555* | *Bacteria* | *Bacteroidetes* | *Bacteroidia* | *Bacteroidales* | *Rikenellaceae* |  |  |
| *2318* | *Bacteria* | *Bacteroidetes* | *Bacteroidia* | *Bacteroidales* | *Porphyromonadaceae* | *Parabacteroides* |  |
| *2244* | *Bacteria* | *Bacteroidetes* | *Bacteroidia* | *Bacteroidales* | *Porphyromonadaceae* | *Parabacteroides* |  |
| *619* | *Bacteria* | *Firmicutes* | *Clostridia* | *Clostridiales* | *Lachnospiraceae* |  |  |
| *2103* | *Bacteria* | *Firmicutes* | *Clostridia* | *Clostridiales* | *Ruminococcaceae* | *Oscillospira* |  |
| *1473* | *Bacteria* | *Bacteroidetes* | *Bacteroidia* | *Bacteroidales* | *Porphyromonadaceae* | *Parabacteroides* |  |
| *3689* | *Bacteria* | *Firmicutes* | *Clostridia* | *Clostridiales* | *Lachnospiraceae* |  |  |
| *403* | *Bacteria* | *Bacteroidetes* | *Bacteroidia* | *Bacteroidales* | *Bacteroidaceae* | *Bacteroides* | *fragilis* |
| *459* | *Bacteria* | *Bacteroidetes* | *Bacteroidia* | *Bacteroidales* | *Bacteroidaceae* | *Bacteroides* |  |
| *1138* | *Bacteria* | *Proteobacteria* | *Gammaproteobacteria* | *Pseudomonadales* | *Pseudomonadaceae* | *Pseudomonas* |  |
| *2453* | *Bacteria* | *Synergistetes* | *Synergistia* | *Synergistales* | *Synergistaceae* | *vadinCA02* |  |
| *1966* | *Bacteria* | *Proteobacteria* | *Gammaproteobacteria* | *Pasteurellales* | *Pasteurellaceae* | *Lonepinella* | *koalarum* |
| *1795* | *Bacteria* | *Verrucomicrobia* | *Verrucomicrobiae* | *Verrucomicrobiales* | *Verrucomicrobiaceae* | *Akkermansia* | *muciniphila* |
| *2308* | *Bacteria* | *Verrucomicrobia* | *Verrucomicrobiae* | *Verrucomicrobiales* | *Verrucomicrobiaceae* | *Akkermansia* |  |
| *402* | *Bacteria* | *Bacteroidetes* | *Bacteroidia* | *Bacteroidales* | *Bacteroidaceae* | *Bacteroides* | *fragilis* |
| *2288* | *Bacteria* | *Firmicutes* | *Clostridia* | *Clostridiales* | *Lachnospiraceae* |  |  |
| *2271* | *Bacteria* | *Bacteroidetes* | *Bacteroidia* | *Bacteroidales* | *Porphyromonadaceae* | *Parabacteroides* |  |
| *1045* | *Bacteria* | *Firmicutes* | *Clostridia* | *Clostridiales* | *Ruminococcaceae* |  |  |
| *1106* | *Bacteria* | *Proteobacteria* | *Gammaproteobacteria* | *Pasteurellales* | *Pasteurellaceae* | *Lonepinella* | *koalarum* |
| *4152* | *Bacteria* | *Firmicutes* | *Clostridia* | *Clostridiales* | *Ruminococcaceae* |  |  |
| *1092* | *Bacteria* | *Firmicutes* | *Clostridia* | *Clostridiales* | *Ruminococcaceae* | *Ruminococcus* | *flavefaciens* |
| *1356* | *Bacteria* | *Firmicutes* | *Clostridia* | *Clostridiales* | *Ruminococcaceae* |  |  |
| *2438* | *Bacteria* | *Fusobacteria* | *Fusobacteriia* | *Fusobacteriales* | *Fusobacteriaceae* | *Fusobacterium* |  |
| *2472* | *Bacteria* | *Proteobacteria* | *Gammaproteobacteria* | *Pasteurellales* | *Pasteurellaceae* | *Lonepinella* | *koalarum* |
| *2490* | *Bacteria* | *Firmicutes* | *Clostridia* | *Clostridiales* | *Ruminococcaceae* |  |  |
| *2446* | *Bacteria* | *Bacteroidetes* | *Bacteroidia* | *Bacteroidales* | *Rikenellaceae* |  |  |
| *2658* | *Bacteria* | *Firmicutes* | *Clostridia* | *Clostridiales* |  |  |  |
| *2250* | *Bacteria* | *Proteobacteria* | *Gammaproteobacteria* | *Pasteurellales* | *Pasteurellaceae* | *Lonepinella* | *koalarum* |
| *1155* | *Bacteria* | *Fusobacteria* | *Fusobacteriia* | *Fusobacteriales* | *Fusobacteriaceae* | *Fusobacterium* |  |
| *1307* | *Bacteria* | *Fusobacteria* | *Fusobacteriia* | *Fusobacteriales* | *Fusobacteriaceae* | *Fusobacterium* |  |
| *2352* | *Bacteria* | *Bacteroidetes* | *Bacteroidia* | *Bacteroidales* | *Rikenellaceae* |  |  |
| *4642* | *Bacteria* | *Bacteroidetes* | *Bacteroidia* | *Bacteroidales* | *Bacteroidaceae* | *Bacteroides* |  |
| *3606* | *Bacteria* | *Firmicutes* | *Clostridia* | *Clostridiales* | *Lachnospiraceae* |  |  |
| *2488* | *Bacteria* | *Synergistetes* | *Synergistia* | *Synergistales* | *Synergistaceae* | *Candidatus Tammella* |  |
| *2476* | *Bacteria* | *Synergistetes* | *Synergistia* | *Synergistales* | *Synergistaceae* |  |  |
| *672* | *Bacteria* | *Bacteroidetes* | *Bacteroidia* | *Bacteroidales* | *Bacteroidaceae* | *Bacteroides* | *fragilis* |
| *2471* | *Bacteria* | *Firmicutes* | *Clostridia* | *Clostridiales* | *Lachnospiraceae* |  |  |
| *555* | *Bacteria* | *Firmicutes* | *Clostridia* | *Clostridiales* |  |  |  |
| *880* | *Bacteria* | *Bacteroidetes* | *Bacteroidia* | *Bacteroidales* | *Bacteroidaceae* | *Bacteroides* | *uniformis* |
| *1960* | *Bacteria* | *Proteobacteria* | *Gammaproteobacteria* | *Pasteurellales* | *Pasteurellaceae* | *Lonepinella* | *koalarum* |
| *2433* | *Bacteria* | *Proteobacteria* | *Deltaproteobacteria* | *Desulfovibrionales* | *Desulfovibrionaceae* |  |  |
| *3565* | *Bacteria* | *Firmicutes* | *Clostridia* | *Clostridiales* | *Ruminococcaceae* | *Ruminococcus* |  |
| *1290* | *Bacteria* | *Bacteroidetes* | *Bacteroidia* | *Bacteroidales* | *Bacteroidaceae* | *Bacteroides* | *fragilis* |
| *2367* | *Bacteria* | *Proteobacteria* | *Deltaproteobacteria* | *Desulfovibrionales* | *Desulfovibrionaceae* |  |  |
| *2279* | *Bacteria* | *Firmicutes* | *Clostridia* | *Clostridiales* | *Lachnospiraceae* |  |  |
| *2393* | *Bacteria* | *Bacteroidetes* | *Bacteroidia* | *Bacteroidales* | *Bacteroidaceae* | *Bacteroides* |  |
| *2457* | *Bacteria* | *Firmicutes* | *Clostridia* | *Clostridiales* | *Lachnospiraceae* |  |  |
| *90* | *Bacteria* | *Firmicutes* | *Clostridia* | *Clostridiales* | *Clostridiaceae* | *Clostridium* |  |
| *1041* | *Bacteria* | *Bacteroidetes* | *Bacteroidia* | *Bacteroidales* | *Bacteroidaceae* | *Bacteroides* | *fragilis* |
| *2164* | *Bacteria* | *Proteobacteria* | *Betaproteobacteria* | *Burkholderiales* | *Comamonadaceae* | *Limnobacter* |  |
| *283* | *Bacteria* | *Proteobacteria* | *Deltaproteobacteria* | *Desulfovibrionales* | *Desulfovibrionaceae* | *Bilophila* |  |
| *2454* | *Bacteria* | *Bacteroidetes* | *Bacteroidia* | *Bacteroidales* | *Bacteroidaceae* |  |  |
| *5304* | *Bacteria* | *Bacteroidetes* | *Bacteroidia* | *Bacteroidales* | *Porphyromonadaceae* | *Parabacteroides* |  |
| *1254* | *Bacteria* | *Firmicutes* | *Clostridia* | *Clostridiales* | *Ruminococcaceae* |  |  |
| *1581* | *Bacteria* | *Proteobacteria* | *Deltaproteobacteria* | *Desulfovibrionales* | *Desulfovibrionaceae* |  |  |
| *2447* | *Bacteria* | *Firmicutes* | *Clostridia* | *Clostridiales* | *Lachnospiraceae* |  |  |
| *962* | *Bacteria* | *Proteobacteria* | *Alphaproteobacteria* | *Rhizobiales* | *Methylobacteriaceae* | *Methylobacterium* | *adhaesivum* |
| *838* | *Bacteria* | *Proteobacteria* | *Betaproteobacteria* | *Burkholderiales* | *Oxalobacteraceae* | *Oxalobacter* | *formigenes* |
| *1444* | *Bacteria* | *Proteobacteria* | *Gammaproteobacteria* | *Enterobacteriales* | *Enterobacteriaceae* |  |  |
| *3190* | *Bacteria* | *Bacteroidetes* | *Bacteroidia* | *Bacteroidales* | *Bacteroidaceae* | *Bacteroides* |  |
| *2302* | *Bacteria* | *Firmicutes* | *Clostridia* | *Clostridiales* |  |  |  |
| *667* | *Bacteria* | *Bacteroidetes* | *Bacteroidia* | *Bacteroidales* | *Porphyromonadaceae* | *Parabacteroides* | *distasonis* |
| *5725* | *Bacteria* | *Firmicutes* | *Clostridia* | *Clostridiales* | *Ruminococcaceae* |  |  |
| *3108* | *Bacteria* | *Bacteroidetes* | *Bacteroidia* | *Bacteroidales* | *Porphyromonadaceae* | *Parabacteroides* |  |
| *1573* | *Bacteria* | *Firmicutes* | *Clostridia* | *Clostridiales* |  |  |  |
| *904* | *Bacteria* | *Firmicutes* | *Clostridia* | *Clostridiales* | *Ruminococcaceae* |  |  |
| *2440* | *Bacteria* | *Bacteroidetes* | *Bacteroidia* | *Bacteroidales* | *Porphyromonadaceae* | *Parabacteroides* |  |
| *703* | *Bacteria* | *Firmicutes* | *Clostridia* | *Clostridiales* | *Ruminococcaceae* |  |  |
| *1541* | *Bacteria* | *Synergistetes* | *Synergistia* | *Synergistales* | *Synergistaceae* |  |  |
| *2375* | *Bacteria* | *Bacteroidetes* | *Bacteroidia* | *Bacteroidales* | *Porphyromonadaceae* | *Parabacteroides* |  |
| *2150* | *Bacteria* | *Bacteroidetes* | *Bacteroidia* | *Bacteroidales* | *Bacteroidaceae* | *Bacteroides* |  |
| *2333* | *Bacteria* | *Proteobacteria* | *Deltaproteobacteria* | *Desulfovibrionales* | *Desulfovibrionaceae* |  |  |
| *439* | *Bacteria* | *Bacteroidetes* | *Bacteroidia* | *Bacteroidales* | *Rikenellaceae* |  |  |
| *942* | *Bacteria* | *Proteobacteria* | *Deltaproteobacteria* | *Desulfovibrionales* | *Desulfovibrionaceae* | *Bilophila* |  |
| *636* | *Bacteria* | *Firmicutes* | *Clostridia* | *Clostridiales* | *Lachnospiraceae* |  |  |
| *671* | *Bacteria* | *Firmicutes* | *Clostridia* | *Clostridiales* | *Lachnospiraceae* |  |  |
| *2491* | *Bacteria* | *Firmicutes* | *Clostridia* | *Clostridiales* | *Ruminococcaceae* | *Oscillospira* |  |
| *5807* | *Bacteria* | *Bacteroidetes* | *Bacteroidia* | *Bacteroidales* | *Bacteroidaceae* | *Bacteroides* |  |
| *1312* | *Bacteria* | *Fusobacteria* | *Fusobacteriia* | *Fusobacteriales* | *Fusobacteriaceae* | *Fusobacterium* |  |
| *1030* | *Bacteria* | *Firmicutes* | *Clostridia* | *Clostridiales* | *Lachnospiraceae* |  |  |
| *25* | *Bacteria* | *Proteobacteria* | *Gammaproteobacteria* | *Pasteurellales* | *Pasteurellaceae* | *Lonepinella* | *koalarum* |
| *1433* | *Bacteria* | *Firmicutes* | *Clostridia* | *Clostridiales* | *Ruminococcaceae* |  |  |
| *3871* | *Bacteria* | *Firmicutes* | *Clostridia* | *Clostridiales* | *Ruminococcaceae* |  |  |
| *1354* | *Bacteria* | *Planctomycetes* | *vadinHA49* | *PeHg47* |  |  |  |
| *1849* | *Bacteria* | *Planctomycetes* | *vadinHA49* | *PeHg47* |  |  |  |
| *1293* | *Bacteria* | *Firmicutes* | *Clostridia* | *Clostridiales* | *Lachnospiraceae* |  |  |
| *1449* | *Bacteria* | *Proteobacteria* | *Deltaproteobacteria* | *Desulfovibrionales* | *Desulfovibrionaceae* | *Bilophila* |  |
| *225* | *Bacteria* | *Firmicutes* | *Clostridia* | *Clostridiales* | *Ruminococcaceae* | *Oscillospira* |  |
| *2263* | *Bacteria* | *Proteobacteria* | *Deltaproteobacteria* | *Desulfovibrionales* | *Desulfovibrionaceae* |  |  |
| *1574* | *Bacteria* | *Bacteroidetes* | *Bacteroidia* | *Bacteroidales* | *Bacteroidaceae* | *Bacteroides* | *uniformis* |
| *2629* | *Bacteria* | *Proteobacteria* | *Deltaproteobacteria* | *Desulfovibrionales* | *Desulfovibrionaceae* |  |  |
| *55* | *Bacteria* | *Bacteroidetes* | *Bacteroidia* | *Bacteroidales* | *Porphyromonadaceae* | *Parabacteroides* | *distasonis* |
| *53* | *Bacteria* | *Planctomycetes* | *vadinHA49* | *PeHg47* |  |  |  |
| *5477* | *Bacteria* | *Proteobacteria* | *Deltaproteobacteria* | *Desulfovibrionales* | *Desulfovibrionaceae* | *Bilophila* |  |
| *3181* | *Bacteria* | *Bacteroidetes* | *Bacteroidia* | *Bacteroidales* | *Rikenellaceae* | *Rikenella* |  |
| *2351* | *Bacteria* | *Bacteroidetes* | *Bacteroidia* | *Bacteroidales* | *Bacteroidaceae* | *Bacteroides* |  |
| *5633* | *Bacteria* | *Proteobacteria* | *Gammaproteobacteria* | *Pasteurellales* | *Pasteurellaceae* | *Lonepinella* | *koalarum* |
| *5803* | *Bacteria* | *Firmicutes* | *Clostridia* | *Clostridiales* | *Lachnospiraceae* |  |  |
| *741* | *Bacteria* | *Proteobacteria* | *Gammaproteobacteria* | *Enterobacteriales* | *Enterobacteriaceae* |  |  |
| *3954* | *Bacteria* | *Firmicutes* | *Clostridia* | *Clostridiales* | *[Mogibacteriaceae]* |  |  |
| *2850* | *Bacteria* | *Firmicutes* | *Clostridia* | *Clostridiales* | *Ruminococcaceae* | *Ruminococcus* |  |
| *1260* | *Bacteria* | *Firmicutes* | *Clostridia* | *Clostridiales* | *Ruminococcaceae* |  |  |
| *5825* | *Bacteria* | *Bacteroidetes* | *Bacteroidia* | *Bacteroidales* | *Bacteroidaceae* | *Bacteroides* | *uniformis* |
| *288* | *Bacteria* | *Proteobacteria* | *Gammaproteobacteria* | *Enterobacteriales* | *Enterobacteriaceae* |  |  |
| *2093* | *Bacteria* | *Firmicutes* | *Clostridia* | *Clostridiales* | *Ruminococcaceae* | *Oscillospira* |  |
| *1850* | *Bacteria* | *Planctomycetes* | *vadinHA49* | *PeHg47* |  |  |  |
| *1871* | *Bacteria* | *Proteobacteria* | *Betaproteobacteria* | *Burkholderiales* | *Oxalobacteraceae* | *Oxalobacter* | *formigenes* |
| *2374* | *Bacteria* | *Firmicutes* | *Clostridia* | *Clostridiales* | *Ruminococcaceae* |  |  |
| *2487* | *Bacteria* | *Firmicutes* | *Clostridia* | *Clostridiales* |  |  |  |
| *2378* | *Bacteria* | *Synergistetes* | *Synergistia* | *Synergistales* | *Synergistaceae* |  |  |
| *2365* | *Bacteria* | *Actinobacteria* | *Coriobacteriia* | *Coriobacteriales* | *Coriobacteriaceae* | *Adlercreutzia* |  |
| *1105* | *Bacteria* | *Firmicutes* | *Clostridia* | *Clostridiales* | *Ruminococcaceae* |  |  |
| *1754* | *Bacteria* | *Proteobacteria* | *Betaproteobacteria* | *Burkholderiales* | *Oxalobacteraceae* |  |  |
| *1014* | *Bacteria* | *Firmicutes* | *Clostridia* | *Clostridiales* |  |  |  |
| *3864* | *Bacteria* | *Bacteroidetes* | *Bacteroidia* | *Bacteroidales* | *Porphyromonadaceae* | *Parabacteroides* |  |
| *2255* | *Bacteria* | *Firmicutes* | *Clostridia* | *Clostridiales* | *Ruminococcaceae* |  |  |
| *2499* | *Bacteria* | *Firmicutes* | *Clostridia* | *Clostridiales* | *Ruminococcaceae* |  |  |
| *1212* | *Bacteria* | *Firmicutes* | *Clostridia* | *Clostridiales* | *Ruminococcaceae* | *Oscillospira* |  |
| *2596* | *Bacteria* | *Bacteroidetes* | *Bacteroidia* | *Bacteroidales* | *Porphyromonadaceae* | *Parabacteroides* |  |
| *151* | *Bacteria* | *Firmicutes* | *Clostridia* | *Clostridiales* | *Ruminococcaceae* |  |  |
| *1970* | *Bacteria* | *Proteobacteria* | *Gammaproteobacteria* | *Enterobacteriales* | *Enterobacteriaceae* |  |  |
| *2316* | *Bacteria* | *Proteobacteria* | *Deltaproteobacteria* | *Desulfovibrionales* | *Desulfovibrionaceae* |  |  |
| *1896* | *Bacteria* | *Firmicutes* | *Clostridia* | *Clostridiales* | *Ruminococcaceae* | *Ruminococcus* | *flavefaciens* |
| *934* | *Bacteria* | *Firmicutes* | *Clostridia* | *Clostridiales* |  |  |  |
| *2362* | *Bacteria* | *Firmicutes* | *Clostridia* | *Clostridiales* | *Lachnospiraceae* |  |  |
| *2483* | *Bacteria* | *Firmicutes* | *Clostridia* | *Clostridiales* |  |  |  |
| *430* | *Bacteria* | *Proteobacteria* | *Deltaproteobacteria* | *Desulfarculales* | *Desulfarculaceae* |  |  |
| *2387* | *Bacteria* | *Firmicutes* | *Clostridia* | *Clostridiales* |  |  |  |
| *2304* | *Bacteria* | *Firmicutes* | *Clostridia* | *Clostridiales* |  |  |  |
| *2429* | *Bacteria* | *Firmicutes* | *Clostridia* | *Clostridiales* | *Ruminococcaceae* |  |  |
| *267* | *Bacteria* | *Firmicutes* | *Clostridia* | *Clostridiales* | *Lachnospiraceae* |  |  |
| *5311* | *Bacteria* | *Firmicutes* | *Clostridia* | *Clostridiales* | *Ruminococcaceae* |  |  |
| *5036* | *Bacteria* | *Firmicutes* | *Clostridia* | *Clostridiales* | *Ruminococcaceae* |  |  |
| *2427* | *Bacteria* | *Proteobacteria* | *Deltaproteobacteria* | *Desulfarculales* | *Desulfarculaceae* |  |  |
| *4764* | *Bacteria* | *Firmicutes* | *Clostridia* | *Clostridiales* | *Ruminococcaceae* |  |  |
| *1054* | *Bacteria* | *Proteobacteria* | *Gammaproteobacteria* | *Enterobacteriales* | *Enterobacteriaceae* |  |  |
| *3313* | *Bacteria* | *Bacteroidetes* | *Bacteroidia* | *Bacteroidales* | *Bacteroidaceae* | *Bacteroides* |  |
| *58* | *Bacteria* | *Firmicutes* | *Clostridia* | *Clostridiales* |  |  |  |
| *3470* | *Bacteria* | *Firmicutes* | *Clostridia* | *Clostridiales* | *Ruminococcaceae* |  |  |
| *953* | *Bacteria* | *Firmicutes* | *Clostridia* | *Clostridiales* |  |  |  |
| *1174* | *Bacteria* | *Firmicutes* | *Clostridia* | *Clostridiales* | *Lachnospiraceae* |  |  |
| *1638* | *Bacteria* | *Proteobacteria* | *Deltaproteobacteria* | *Desulfovibrionales* | *Desulfovibrionaceae* |  |  |
| *2247* | *Bacteria* | *Cyanobacteria* | *4C0d-2* | *YS2* |  |  |  |
| *4170* | *Bacteria* | *Actinobacteria* | *Coriobacteriia* | *Coriobacteriales* | *Coriobacteriaceae* |  |  |
| *3129* | *Bacteria* | *Firmicutes* | *Clostridia* | *Clostridiales* |  |  |  |
| *593* | *Bacteria* | *Proteobacteria* | *Gammaproteobacteria* | *Enterobacteriales* | *Enterobacteriaceae* |  |  |
| *1919* | *Bacteria* | *Fusobacteria* | *Fusobacteriia* | *Fusobacteriales* | *Fusobacteriaceae* | *Fusobacterium* |  |
| *4546* | *Bacteria* | *Synergistetes* | *Synergistia* | *Synergistales* | *Synergistaceae* |  |  |
| *3628* | *Bacteria* | *Planctomycetes* | *vadinHA49* | *PeHg47* |  |  |  |
| *2344* | *Bacteria* | *Firmicutes* | *Clostridia* | *Clostridiales* |  |  |  |
| *330* | *Bacteria* | *Bacteroidetes* | *Bacteroidia* | *Bacteroidales* | *Rikenellaceae* |  |  |
| *4082* | *Bacteria* | *Firmicutes* | *Clostridia* | *Clostridiales* | *Lachnospiraceae* |  |  |
| *5105* | *Bacteria* | *Firmicutes* | *Clostridia* | *Clostridiales* | *Ruminococcaceae* |  |  |
| *1789* | *Bacteria* | *Verrucomicrobia* | *Verrucomicrobiae* | *Verrucomicrobiales* | *Verrucomicrobiaceae* | *Akkermansia* | *muciniphila* |
| *2286* | *Bacteria* | *Bacteroidetes* | *Bacteroidia* | *Bacteroidales* | *[Odoribacteraceae]* | *Odoribacter* |  |
| *5187* | *Bacteria* | *Bacteroidetes* | *Bacteroidia* | *Bacteroidales* | *Porphyromonadaceae* | *Parabacteroides* |  |
| *1839* | *Bacteria* | *Firmicutes* | *Clostridia* | *Clostridiales* | *Veillonellaceae* |  |  |
| *1418* | *Bacteria* | *Proteobacteria* | *Gammaproteobacteria* | *Pseudomonadales* | *Pseudomonadaceae* | *Pseudomonas* |  |
| *788* | *Bacteria* | *Firmicutes* | *Clostridia* | *Clostridiales* | *Lachnospiraceae* |  |  |
| *1517* | *Bacteria* | *Proteobacteria* | *Deltaproteobacteria* | *Desulfovibrionales* | *Desulfovibrionaceae* |  |  |
| *5494* | *Bacteria* | *Deferribacteres* | *Deferribacteres* | *Deferribacterales* | *Deferribacteraceae* | *Mucispirillum* |  |
| *5354* | *Bacteria* | *Bacteroidetes* | *Bacteroidia* | *Bacteroidales* | *Rikenellaceae* |  |  |
| *1003* | *Bacteria* | *Bacteroidetes* | *Bacteroidia* | *Bacteroidales* | *Bacteroidaceae* | *Bacteroides* |  |
| *2426* | *Bacteria* | *Firmicutes* | *Clostridia* | *Clostridiales* | *Ruminococcaceae* |  |  |
| *2459* | *Bacteria* | *Firmicutes* | *Clostridia* | *Clostridiales* | *Ruminococcaceae* |  |  |
| *91* | *Bacteria* | *Bacteroidetes* | *Bacteroidia* | *Bacteroidales* | *Bacteroidaceae* | *Bacteroides* | *fragilis* |
| *2357* | *Bacteria* | *Bacteroidetes* | *Bacteroidia* | *Bacteroidales* | *Porphyromonadaceae* | *Parabacteroides* |  |
| *5451* | *Bacteria* | *Bacteroidetes* | *Bacteroidia* | *Bacteroidales* | *Bacteroidaceae* | *Bacteroides* |  |
| *2290* | *Bacteria* | *Bacteroidetes* | *Bacteroidia* | *Bacteroidales* | *Bacteroidaceae* | *Bacteroides* |  |
| *2452* | *Bacteria* | *Firmicutes* | *Clostridia* | *Clostridiales* | *Ruminococcaceae* |  |  |
| *1160* | *Bacteria* | *Firmicutes* | *Clostridia* | *Clostridiales* | *Ruminococcaceae* | *Oscillospira* |  |
| *4144* | *Bacteria* | *Firmicutes* | *Clostridia* | *Clostridiales* | *Ruminococcaceae* |  |  |
| *2493* | *Bacteria* | *Synergistetes* | *Synergistia* | *Synergistales* | *Synergistaceae* |  |  |
| *3964* | *Bacteria* | *Firmicutes* | *Clostridia* | *Clostridiales* | *Ruminococcaceae* |  |  |
| *134* | *Bacteria* | *Proteobacteria* | *Betaproteobacteria* | *Burkholderiales* | *Alcaligenaceae* | *Sutterella* |  |
| *2253* | *Bacteria* | *Firmicutes* | *Clostridia* | *Clostridiales* |  |  |  |
| *996* | *Bacteria* | *Firmicutes* | *Clostridia* | *Clostridiales* | *Ruminococcaceae* |  |  |
| *3677* | *Bacteria* | *Firmicutes* | *Clostridia* | *Clostridiales* |  |  |  |
| *1020* | *Bacteria* | *Firmicutes* | *Clostridia* | *Clostridiales* | *Ruminococcaceae* |  |  |
| *2797* | *Bacteria* | *Firmicutes* | *Clostridia* | *Clostridiales* | *Ruminococcaceae* |  |  |
| *2291* | *Bacteria* | *Bacteroidetes* | *Bacteroidia* | *Bacteroidales* | *Porphyromonadaceae* | *Parabacteroides* |  |
| *311* | *Bacteria* | *Firmicutes* | *Clostridia* | *Clostridiales* | *Ruminococcaceae* | *Ruminococcus* |  |
| *732* | *Bacteria* | *Proteobacteria* | *Gammaproteobacteria* | *Enterobacteriales* | *Enterobacteriaceae* |  |  |
| *2389* | *Bacteria* | *Firmicutes* | *Clostridia* | *Clostridiales* |  |  |  |
| *5629* | *Bacteria* | *Firmicutes* | *Clostridia* | *Clostridiales* | *Ruminococcaceae* |  |  |
| *4138* | *Bacteria* | *Firmicutes* | *Clostridia* | *Clostridiales* |  |  |  |
| *1536* | *Bacteria* | *Firmicutes* | *Clostridia* | *Clostridiales* |  |  |  |
| *1880* | *Bacteria* | *Proteobacteria* | *Gammaproteobacteria* | *Enterobacteriales* | *Enterobacteriaceae* | *Erwinia* |  |
| *2285* | *Bacteria* | *Firmicutes* | *Clostridia* | *Clostridiales* | *Lachnospiraceae* |  |  |
| *2439* | *Bacteria* | *Firmicutes* | *Clostridia* | *Clostridiales* |  |  |  |
| *733* | *Bacteria* | *Firmicutes* | *Clostridia* | *Clostridiales* | *Ruminococcaceae* |  |  |
| *2353* | *Bacteria* | *Firmicutes* | *Clostridia* | *Clostridiales* | *Lachnospiraceae* |  |  |
| *2305* | *Bacteria* | *Firmicutes* | *Clostridia* | *Clostridiales* | *Lachnospiraceae* |  |  |
| *415* | *Bacteria* | *Firmicutes* | *Clostridia* | *Clostridiales* | *Ruminococcaceae* | *Ruminococcus* | *flavefaciens* |
| *2392* | *Bacteria* | *Proteobacteria* | *Betaproteobacteria* | *Rhodocyclales* | *Rhodocyclaceae* |  |  |
| *2349* | *Bacteria* | *Bacteroidetes* | *Bacteroidia* | *Bacteroidales* | *Porphyromonadaceae* | *Parabacteroides* |  |
| *937* | *Bacteria* | *Firmicutes* | *Clostridia* | *Clostridiales* | *Ruminococcaceae* | *Ruminococcus* |  |
| *4583* | *Bacteria* | *Proteobacteria* | *Gammaproteobacteria* | *Pasteurellales* | *Pasteurellaceae* | *Lonepinella* | *koalarum* |
| *1677* | *Bacteria* | *Bacteroidetes* | *Bacteroidia* | *Bacteroidales* | *Rikenellaceae* |  |  |
| *3661* | *Bacteria* | *Firmicutes* | *Clostridia* | *Clostridiales* |  |  |  |
| *1135* | *Bacteria* | *Firmicutes* | *Clostridia* | *Clostridiales* | *Ruminococcaceae* | *Oscillospira* |  |
| *2348* | *Bacteria* | *Bacteroidetes* | *Bacteroidia* | *Bacteroidales* | *Porphyromonadaceae* | *Parabacteroides* |  |
| *2504* | *Bacteria* | *Firmicutes* | *Erysipelotrichi* | *Erysipelotrichales* | *Erysipelotrichaceae* | *Coprobacillus* |  |
| *1183* | *Bacteria* | *Synergistetes* | *Synergistia* | *Synergistales* | *Synergistaceae* |  |  |
| *1841* | *Bacteria* | *Bacteroidetes* | *Bacteroidia* | *Bacteroidales* | *Porphyromonadaceae* | *Parabacteroides* |  |
| *800* | *Bacteria* | *Firmicutes* | *Clostridia* | *Clostridiales* | *Lachnospiraceae* |  |  |
| *2388* | *Bacteria* | *Proteobacteria* | *Deltaproteobacteria* | *Desulfovibrionales* | *Desulfovibrionaceae* |  |  |
| *1584* | *Bacteria* | *Bacteroidetes* | *Bacteroidia* | *Bacteroidales* | *[Odoribacteraceae]* | *Odoribacter* |  |
| *5517* | *Bacteria* | *Firmicutes* | *Clostridia* | *Clostridiales* | *Lachnospiraceae* |  |  |
| *704* | *Bacteria* | *Firmicutes* | *Clostridia* | *Clostridiales* | *Lachnospiraceae* |  |  |
| *2046* | *Bacteria* | *Bacteroidetes* | *Bacteroidia* | *Bacteroidales* | *Bacteroidaceae* | *Bacteroides* |  |
| *938* | *Bacteria* | *Planctomycetes* | *vadinHA49* | *PeHg47* |  |  |  |
| *5285* | *Bacteria* | *Firmicutes* | *Clostridia* | *Clostridiales* | *Ruminococcaceae* | *Ruminococcus* | *flavefaciens* |
| *2264* | *Bacteria* | *Bacteroidetes* | *Bacteroidia* | *Bacteroidales* | *Porphyromonadaceae* | *Parabacteroides* | *distasonis* |
| *1730* | *Bacteria* | *Bacteroidetes* | *Bacteroidia* | *Bacteroidales* | *Rikenellaceae* | *Rikenella* |  |
| *5041* | *Bacteria* | *Bacteroidetes* | *Bacteroidia* | *Bacteroidales* | *Porphyromonadaceae* | *Parabacteroides* |  |
| *1577* | *Bacteria* | *Firmicutes* | *Clostridia* | *Clostridiales* | *Ruminococcaceae* | *Oscillospira* |  |
| *4753* | *Bacteria* | *Synergistetes* | *Synergistia* | *Synergistales* | *Synergistaceae* |  |  |
| *4197* | *Bacteria* | *Firmicutes* | *Clostridia* | *Clostridiales* | *Lachnospiraceae* |  |  |
| *4116* | *Bacteria* | *Firmicutes* | *Clostridia* | *Clostridiales* | *Lachnospiraceae* |  |  |
| *496* | *Bacteria* | *Firmicutes* | *Clostridia* | *Clostridiales* | *Lachnospiraceae* |  |  |
| *2497* | *Bacteria* | *Bacteroidetes* | *Bacteroidia* | *Bacteroidales* | *Bacteroidaceae* | *Bacteroides* |  |
| *5840* | *Bacteria* | *Firmicutes* | *Clostridia* | *Clostridiales* | *Lachnospiraceae* |  |  |
| *3392* | *Bacteria* | *Firmicutes* | *Clostridia* | *Clostridiales* |  |  |  |
| *1383* | *Bacteria* | *Proteobacteria* | *Gammaproteobacteria* | *Pasteurellales* | *Pasteurellaceae* | *Lonepinella* | *koalarum* |
| *5077* | *Bacteria* | *Firmicutes* | *Clostridia* | *Clostridiales* | *Lachnospiraceae* |  |  |
| *257* | *Bacteria* | *Firmicutes* | *Clostridia* | *Clostridiales* | *Ruminococcaceae* |  |  |
| *2359* | *Bacteria* | *Bacteroidetes* | *Bacteroidia* | *Bacteroidales* | *Bacteroidaceae* | *Bacteroides* |  |
| *2489* | *Bacteria* | *Synergistetes* | *Synergistia* | *Synergistales* | *Synergistaceae* |  |  |
| *2498* | *Bacteria* | *Firmicutes* | *Clostridia* | *Clostridiales* | *Ruminococcaceae* |  |  |
| *1991* | *Bacteria* | *Firmicutes* | *Clostridia* | *Clostridiales* | *Ruminococcaceae* |  |  |
| *2289* | *Bacteria* | *Firmicutes* | *Clostridia* | *Clostridiales* | *Ruminococcaceae* | *Ruminococcus* |  |
| *3533* | *Bacteria* | *Firmicutes* | *Clostridia* | *Clostridiales* | *Lachnospiraceae* |  |  |
| *422* | *Bacteria* | *Proteobacteria* | *Gammaproteobacteria* | *Enterobacteriales* | *Enterobacteriaceae* |  |  |
| *3978* | *Bacteria* | *Firmicutes* | *Clostridia* | *Clostridiales* | *Lachnospiraceae* |  |  |
| *2377* | *Bacteria* | *Firmicutes* | *Clostridia* | *Clostridiales* | *Ruminococcaceae* |  |  |
| *1176* | *Bacteria* | *Firmicutes* | *Clostridia* | *Clostridiales* | *Ruminococcaceae* |  |  |
| *956* | *Bacteria* | *Bacteroidetes* | *Bacteroidia* | *Bacteroidales* | *Porphyromonadaceae* | *Parabacteroides* | *distasonis* |
| *970* | *Bacteria* | *Firmicutes* | *Clostridia* | *Clostridiales* | *Ruminococcaceae* | *Ruminococcus* |  |
| *2300* | *Bacteria* | *Bacteroidetes* | *Bacteroidia* | *Bacteroidales* | *Bacteroidaceae* | *Bacteroides* |  |
| *2400* | *Bacteria* | *Bacteroidetes* | *Bacteroidia* | *Bacteroidales* | *Porphyromonadaceae* | *Parabacteroides* |  |
| *4497* | *Bacteria* | *Firmicutes* | *Clostridia* | *Clostridiales* | *Ruminococcaceae* | *Oscillospira* |  |
| *4271* | *Bacteria* | *Firmicutes* | *Clostridia* | *Clostridiales* | *Ruminococcaceae* | *Ruminococcus* | *flavefaciens* |
| *2311* | *Bacteria* | *Proteobacteria* | *Deltaproteobacteria* | *Desulfovibrionales* | *Desulfovibrionaceae* |  |  |
| *2384* | *Bacteria* | *Proteobacteria* | *Betaproteobacteria* | *Rhodocyclales* | *Rhodocyclaceae* |  |  |
| *425* | *Bacteria* | *Firmicutes* | *Clostridia* | *Clostridiales* | *Lachnospiraceae* |  |  |
| *3679* | *Bacteria* | *Proteobacteria* | *Deltaproteobacteria* | *Desulfarculales* | *Desulfarculaceae* |  |  |
| *4744* | *Bacteria* | *Proteobacteria* | *Deltaproteobacteria* | *Desulfarculales* | *Desulfarculaceae* |  |  |
| *4077* | *Bacteria* | *Proteobacteria* | *Deltaproteobacteria* | *Desulfovibrionales* | *Desulfovibrionaceae* |  |  |
| *1233* | *Bacteria* | *Firmicutes* | *Clostridia* | *Clostridiales* | *Ruminococcaceae* |  |  |
| *5631* | *Bacteria* | *Proteobacteria* | *Deltaproteobacteria* |  |  |  |  |
| *1933* | *Bacteria* | *Firmicutes* | *Clostridia* | *Clostridiales* | *Clostridiaceae* | *Clostridium* |  |
| *872* | *Bacteria* | *Bacteroidetes* | *Bacteroidia* | *Bacteroidales* | *Bacteroidaceae* | *Bacteroides* | *ovatus* |
| *1662* | *Bacteria* | *Firmicutes* | *Clostridia* | *Clostridiales* | *Lachnospiraceae* |  |  |
| *2380* | *Bacteria* | *Bacteroidetes* | *Bacteroidia* | *Bacteroidales* | *Porphyromonadaceae* | *Parabacteroides* |  |
| *1613* | *Bacteria* | *Proteobacteria* | *Gammaproteobacteria* | *Enterobacteriales* | *Enterobacteriaceae* |  |  |
| *1242* | *Bacteria* | *Bacteroidetes* | *Bacteroidia* | *Bacteroidales* | *Porphyromonadaceae* | *Parabacteroides* |  |
| *1034* | *Bacteria* | *Proteobacteria* | *Gammaproteobacteria* | *Enterobacteriales* | *Enterobacteriaceae* | *Citrobacter* |  |
| *560* | *Bacteria* | *Firmicutes* | *Clostridia* | *Clostridiales* | *Ruminococcaceae* | *Ruminococcus* | *flavefaciens* |
| *658* | *Bacteria* | *Bacteroidetes* | *Bacteroidia* | *Bacteroidales* | *Bacteroidaceae* | *Bacteroides* |  |
| *2251* | *Bacteria* | *Firmicutes* | *Clostridia* | *Clostridiales* |  |  |  |
| *2254* | *Bacteria* | *Firmicutes* | *Clostridia* | *Clostridiales* | *Lachnospiraceae* |  |  |
| *2242* | *Bacteria* | *Firmicutes* | *Clostridia* | *Clostridiales* | *Ruminococcaceae* |  |  |
| *197* | *Bacteria* | *Firmicutes* | *Clostridia* | *Clostridiales* | *Ruminococcaceae* | *Ruminococcus* | *flavefaciens* |
| *1773* | *Bacteria* | *Bacteroidetes* | *Bacteroidia* | *Bacteroidales* | *Porphyromonadaceae* | *Parabacteroides* |  |
| *1727* | *Bacteria* | *Firmicutes* | *Clostridia* | *Clostridiales* | *Ruminococcaceae* |  |  |
| *3307* | *Bacteria* | *Firmicutes* | *Clostridia* | *Clostridiales* | *Ruminococcaceae* |  |  |
| *3072* | *Bacteria* | *Firmicutes* | *Clostridia* | *Clostridiales* | *Lachnospiraceae* |  |  |
| *634* | *Bacteria* | *Bacteroidetes* | *Bacteroidia* | *Bacteroidales* | *Porphyromonadaceae* | *Parabacteroides* |  |
| *806* | *Bacteria* | *Proteobacteria* | *Gammaproteobacteria* | *Pasteurellales* | *Pasteurellaceae* | *Lonepinella* | *koalarum* |
| *5353* | *Bacteria* | *Firmicutes* | *Clostridia* | *Clostridiales* | *Ruminococcaceae* |  |  |
| *2409* | *Bacteria* | *Firmicutes* | *Clostridia* | *Clostridiales* | *Ruminococcaceae* |  |  |
| *5438* | *Bacteria* | *Firmicutes* | *Clostridia* | *Clostridiales* | *Ruminococcaceae* | *Oscillospira* |  |
| *89* | *Bacteria* | *Bacteroidetes* | *Bacteroidia* | *Bacteroidales* | *Bacteroidaceae* | *Bacteroides* |  |
| *5462* | *Bacteria* | *Fusobacteria* | *Fusobacteriia* | *Fusobacteriales* | *Fusobacteriaceae* | *Fusobacterium* |  |
| *4654* | *Bacteria* | *Proteobacteria* | *Gammaproteobacteria* | *Pasteurellales* | *Pasteurellaceae* | *Lonepinella* | *koalarum* |
| *3337* | *Bacteria* | *Firmicutes* | *Clostridia* | *Clostridiales* | *Lachnospiraceae* | *[Ruminococcus]* | *gnavus* |
| *2328* | *Bacteria* | *Cyanobacteria* | *4C0d-2* | *YS2* |  |  |  |
| *773* | *Bacteria* | *Firmicutes* | *Clostridia* | *Clostridiales* | *Lachnospiraceae* |  |  |
| *1603* | *Bacteria* | *Firmicutes* | *Clostridia* | *Clostridiales* |  |  |  |
| *1262* | *Bacteria* | *Firmicutes* | *Clostridia* | *Clostridiales* | *Ruminococcaceae* | *Oscillospira* |  |
| *2468* | *Bacteria* | *Bacteroidetes* | *Bacteroidia* | *Bacteroidales* | *Rikenellaceae* |  |  |
| *2281* | *Bacteria* | *Bacteroidetes* | *Bacteroidia* | *Bacteroidales* | *Porphyromonadaceae* | *Parabacteroides* | *distasonis* |
| *2445* | *Bacteria* | *Bacteroidetes* | *Bacteroidia* | *Bacteroidales* | *Rikenellaceae* |  |  |
| *1363* | *Bacteria* | *Proteobacteria* | *Gammaproteobacteria* | *Enterobacteriales* | *Enterobacteriaceae* |  |  |
| *3873* | *Bacteria* | *Deferribacteres* | *Deferribacteres* | *Deferribacterales* | *Deferribacteraceae* | *Mucispirillum* |  |
| *2268* | *Bacteria* | *Firmicutes* | *Clostridia* | *Clostridiales* | *Ruminococcaceae* | *Oscillospira* |  |
| *5546* | *Bacteria* | *Bacteroidetes* | *Bacteroidia* | *Bacteroidales* | *Bacteroidaceae* | *Bacteroides* |  |
| *2998* | *Bacteria* | *Synergistetes* | *Synergistia* | *Synergistales* | *Synergistaceae* | *Synergistes* |  |
| *1111* | *Bacteria* | *Bacteroidetes* | *Bacteroidia* | *Bacteroidales* | *Porphyromonadaceae* | *Parabacteroides* | *distasonis* |
| *5650* | *Bacteria* | *Firmicutes* | *Clostridia* | *Clostridiales* | *Christensenellaceae* |  |  |
| *3959* | *Bacteria* | *Bacteroidetes* | *Bacteroidia* | *Bacteroidales* | *Rikenellaceae* |  |  |
| *2451* | *Bacteria* | *Cyanobacteria* | *4C0d-2* | *YS2* |  |  |  |
| *1810* | *Bacteria* | *Firmicutes* | *Clostridia* | *Clostridiales* | *Ruminococcaceae* |  |  |
| *3617* | *Bacteria* | *Firmicutes* | *Clostridia* | *Clostridiales* | *Ruminococcaceae* |  |  |
| *1918* | *Bacteria* | *Proteobacteria* | *Gammaproteobacteria* | *Enterobacteriales* | *Enterobacteriaceae* |  |  |
| *5260* | *Bacteria* | *Firmicutes* | *Clostridia* | *Clostridiales* |  |  |  |
| *1132* | *Bacteria* | *Firmicutes* | *Clostridia* | *Clostridiales* | *Ruminococcaceae* | *Ruminococcus* |  |
| *2422* | *Bacteria* | *Firmicutes* | *Clostridia* | *Clostridiales* | *Lachnospiraceae* |  |  |
| *1365* | *Bacteria* | *Proteobacteria* | *Deltaproteobacteria* | *Desulfovibrionales* | *Desulfovibrionaceae* |  |  |
| *2092* | *Bacteria* | *Proteobacteria* | *Gammaproteobacteria* | *Enterobacteriales* | *Enterobacteriaceae* |  |  |
| *2500* | *Bacteria* | *Firmicutes* | *Clostridia* | *Clostridiales* | *Veillonellaceae* |  |  |
| *4035* | *Bacteria* | *Firmicutes* | *Clostridia* | *Clostridiales* | *Ruminococcaceae* |  |  |
| *3198* | *Bacteria* | *Firmicutes* | *Clostridia* | *Clostridiales* | *Ruminococcaceae* | *Ruminococcus* |  |
| *4853* | *Bacteria* | *Bacteroidetes* | *Bacteroidia* | *Bacteroidales* | *Bacteroidaceae* | *Bacteroides* |  |
| *2294* | *Bacteria* | *Bacteroidetes* | *Bacteroidia* | *Bacteroidales* | *Porphyromonadaceae* | *Parabacteroides* | *distasonis* |
| *3442* | *Bacteria* | *Bacteroidetes* | *Bacteroidia* | *Bacteroidales* | *Porphyromonadaceae* | *Parabacteroides* | *distasonis* |
| *4729* | *Bacteria* | *Bacteroidetes* | *Bacteroidia* | *Bacteroidales* | *Porphyromonadaceae* | *Parabacteroides* |  |
| *2391* | *Bacteria* | *Firmicutes* | *Clostridia* | *Clostridiales* |  |  |  |
| *2114* | *Bacteria* | *Bacteroidetes* | *Bacteroidia* | *Bacteroidales* | *Bacteroidaceae* | *Bacteroides* |  |
| *2405* | *Bacteria* | *Bacteroidetes* | *Bacteroidia* | *Bacteroidales* | *Porphyromonadaceae* |  |  |
| *3856* | *Bacteria* | *Bacteroidetes* | *Bacteroidia* | *Bacteroidales* | *Porphyromonadaceae* | *Parabacteroides* |  |
| *929* | *Bacteria* | *Proteobacteria* | *Deltaproteobacteria* | *Desulfovibrionales* | *Desulfovibrionaceae* | *Bilophila* |  |
| *3610* | *Bacteria* | *Bacteroidetes* | *Bacteroidia* | *Bacteroidales* | *Bacteroidaceae* | *Bacteroides* |  |
| *2350* | *Bacteria* | *Firmicutes* | *Clostridia* | *Clostridiales* | *Ruminococcaceae* |  |  |
| *1765* | *Bacteria* | *Proteobacteria* | *Gammaproteobacteria* | *Enterobacteriales* | *Enterobacteriaceae* |  |  |
| *5548* | *Bacteria* | *Firmicutes* | *Clostridia* | *Clostridiales* | *Ruminococcaceae* |  |  |
| *15* | *Bacteria* | *Firmicutes* | *Clostridia* | *Clostridiales* | *Lachnospiraceae* |  |  |
| *2395* | *Bacteria* | *Bacteroidetes* | *Bacteroidia* | *Bacteroidales* | *Bacteroidaceae* | *Bacteroides* |  |
| *2315* | *Bacteria* | *Firmicutes* | *Clostridia* | *Clostridiales* | *Veillonellaceae* |  |  |
| *2147* | *Bacteria* | *Proteobacteria* | *Gammaproteobacteria* | *Pasteurellales* | *Pasteurellaceae* | *Lonepinella* | *koalarum* |
| *2756* | *Bacteria* | *Proteobacteria* | *Gammaproteobacteria* | *Enterobacteriales* | *Enterobacteriaceae* |  |  |
| *2419* | *Bacteria* | *Firmicutes* | *Clostridia* | *Clostridiales* | *Ruminococcaceae* |  |  |
| *5089* | *Bacteria* | *Firmicutes* | *Clostridia* | *Clostridiales* | *Lachnospiraceae* |  |  |
| *2345* | *Bacteria* | *Bacteroidetes* | *Bacteroidia* | *Bacteroidales* | *[Paraprevotellaceae]* | *Paraprevotella* |  |
| *2342* | *Bacteria* | *Bacteroidetes* | *Bacteroidia* | *Bacteroidales* | *Porphyromonadaceae* | *Parabacteroides* |  |
| *1809* | *Bacteria* | *Proteobacteria* | *Gammaproteobacteria* | *Enterobacteriales* | *Enterobacteriaceae* |  |  |
| *4670* | *Bacteria* | *Bacteroidetes* | *Bacteroidia* | *Bacteroidales* | *Rikenellaceae* |  |  |
| *244* | *Bacteria* | *Proteobacteria* | *Gammaproteobacteria* | *Enterobacteriales* | *Enterobacteriaceae* |  |  |
| *1726* | *Bacteria* | *Proteobacteria* | *Gammaproteobacteria* | *Enterobacteriales* | *Enterobacteriaceae* |  |  |
| *2361* | *Bacteria* | *Bacteroidetes* | *Bacteroidia* | *Bacteroidales* | *Porphyromonadaceae* | *Parabacteroides* |  |
| *1957* | *Bacteria* | *Proteobacteria* | *Gammaproteobacteria* | *Pseudomonadales* | *Moraxellaceae* | *Acinetobacter* |  |
| *167* | *Bacteria* | *Bacteroidetes* | *Bacteroidia* | *Bacteroidales* | *Porphyromonadaceae* | *Parabacteroides* |  |
| *2329* | *Bacteria* | *Bacteroidetes* | *Bacteroidia* | *Bacteroidales* | *Porphyromonadaceae* | *Parabacteroides* |  |
| *2336* | *Bacteria* | *Firmicutes* | *Clostridia* | *Clostridiales* | *Ruminococcaceae* |  |  |
| *3554* | *Bacteria* | *Firmicutes* | *Clostridia* | *Clostridiales* | *Ruminococcaceae* |  |  |
| *2169* | *Bacteria* | *Firmicutes* | *Clostridia* | *Clostridiales* | *Lachnospiraceae* |  |  |
| *3097* | *Bacteria* | *Proteobacteria* | *Deltaproteobacteria* | *Desulfovibrionales* | *Desulfovibrionaceae* |  |  |
| *1147* | *Bacteria* | *Firmicutes* | *Clostridia* | *Clostridiales* | *Lachnospiraceae* |  |  |
| *487* | *Bacteria* | *Firmicutes* | *Clostridia* | *Clostridiales* | *Lachnospiraceae* |  |  |
| *902* | *Bacteria* | *Firmicutes* | *Clostridia* | *Clostridiales* | *Lachnospiraceae* |  |  |
| *981* | *Bacteria* | *Firmicutes* | *Clostridia* | *Clostridiales* | *Ruminococcaceae* | *Oscillospira* |  |
| *1209* | *Bacteria* | *Proteobacteria* | *Gammaproteobacteria* | *Pasteurellales* | *Pasteurellaceae* | *Haemophilus* |  |
| *2402* | *Bacteria* | *Firmicutes* | *Clostridia* | *Clostridiales* | *Ruminococcaceae* |  |  |
| *4984* | *Bacteria* | *Bacteroidetes* | *Bacteroidia* | *Bacteroidales* | *Porphyromonadaceae* | *Parabacteroides* |  |
| *2888* | *Bacteria* | *Bacteroidetes* | *Bacteroidia* | *Bacteroidales* | *Porphyromonadaceae* | *Parabacteroides* |  |
| *3668* | *Bacteria* | *Proteobacteria* | *Gammaproteobacteria* | *Pasteurellales* | *Pasteurellaceae* | *Lonepinella* | *koalarum* |
| *3449* | *Bacteria* | *Proteobacteria* | *Deltaproteobacteria* | *Desulfovibrionales* | *Desulfovibrionaceae* | *Bilophila* |  |
| *4183* | *Bacteria* | *Bacteroidetes* | *Bacteroidia* | *Bacteroidales* | *Bacteroidaceae* | *Bacteroides* | *ovatus* |
| *2776* | *Bacteria* | *Bacteroidetes* | *Bacteroidia* | *Bacteroidales* | *Bacteroidaceae* | *Bacteroides* | *fragilis* |
| *1122* | *Bacteria* | *Planctomycetes* | *vadinHA49* | *PeHg47* |  |  |  |
